# Atypical Expression of Smooth Muscle Markers and Co-activators and Their Regulation in Rheumatic Aortic and Calcified Bicuspid Valves

**DOI:** 10.3389/fcvm.2022.793666

**Published:** 2022-03-17

**Authors:** Najma Latif, Padmini Sarathchandra, Ann McCormack, Magdi H. Yacoub, Adrian H. Chester

**Affiliations:** ^1^Heart Science Centre, Magdi Yacoub Institute, Harefield, United Kingdom; ^2^National Heart and Lung Institute, Imperial College London, London, United Kingdom

**Keywords:** rheumatic, bicuspid, valve, endothelial cells, interstitial cells

## Abstract

**Objective:**

We have previously reported that human calcified aortic cusps have abundant expression of smooth muscle (SM) markers and co-activators. We hypothesised that cells in bicuspid aortic valve (BAV) cusps and those affected by rheumatic heart valve (RHV) disease may follow a similar phenotypic transition into smooth muscle cells, a process that could be regulated by transforming growth factors (TGFs).

**Aims:**

Cusps from eight patients with BAV and seven patients with RHV were analysed for early and late SM markers and regulators of SM gene expression by immunocytochemistry and compared to healthy aortic valves from 12 unused heart valve donors. The ability of TGFs to induce these markers in valve endothelial cells (VECs) on two substrates was assessed.

**Results:**

In total, 7 out of 8 BAVs and all the RHVs showed an increased and atypical expression of early and late SM markers α-SMA, calponin, SM22 and SM-myosin. The SM marker co-activators were aberrantly expressed in six of the BAV and six of the RHV, in a similar regional pattern to the expression of SM markers. Additionally, regions of VECs, and endothelial cells lining the vessels within the cusps were found to be positive for SM markers and co-activators in three BAV and six RHV. Both BAVs and RHVs were significantly thickened and HIF1α expression was prominent in four BAVs and one RHV. The ability of TGFβs to induce the expression of SM markers and myocardin was greater in VECs cultured on fibronectin than on gelatin. Fibronectin was shown to be upregulated in BAVs and RHVs, within the cusps as well as in the basement membrane.

**Conclusion:**

Bicuspid aortic valves and RHVs expressed increased numbers of SM marker-positive VICs and VECs. Concomittantly, these cells expressed MRTF-A and myocardin, key regulators of SM gene expression. TGFβ1 was able to preferentially upregulate SM markers and myocardin in VECs on fibronectin, and fibronectin was found to be upregulated in BAVs and RHVs. These findings suggest a role of VEC as a source of cells that express SM cell markers in BAVs and RHVs. The similarity between SM marker expression in BAVs and RHVs with our previous study with cusps from patients with aortic stenosis suggests the existance of a common pathological pathway between these different pathologies.

## Highlights

- Increased and aberrant expression of SM markers and co-activators was observed in all the BAV and RHV cusps.
- The pattern of expression was diffuse in the RHD and localised around calcified areas in BAV.
- RHV showed expression of SM markers and SM co-activators in both VICs, VECs and vessels, BAV showed expression in VICs and a smaller percentage of VECs.
- Strong HIF1α expression was present in 4 of the BAV and 2 RHV and the pattern of expression did not correlate with SM markers.
- TGFβ1 did not significantly influence SM expression by VECs on gelatin.
- Combined fibronectin coating and TGFβ1 treatment resulted in increased expression of SM markers and myocardin by VECs.
- BAV and RHV cusps demonstrated enhanced expression of fibronectin.

## Introduction

Bicuspid aortic valve (BAV) has an estimated prevalence of 0.5–2% (1), a male predominance of about 3:1 and is a developmental aberration (2). The valves usually exhibit normal function at birth, however, the development of valve disease is expedited and typically develops at a much younger age than in people with tricuspid aortic valves. BAV complications include moderate to severe aortic regurgitation (prevalence 13–30%), moderate to severe aortic stenosis (12–37%) and aortic dilatation (20–40%) with lower incidences of endocarditis (3). BAVs experience abnormal flow patterns compared to tricuspid valves resulting in higher mechanical stresses on the cusps and together with inflammation and increased lipid deposition, the process of mineralisation is initiated. It is known that BAV has a heritable nature, however, the genetic causes are still unravelling with mutations in NOTCH1 being implicated (4) and a potential role for GATA5 (5).

Rheumatic heart valve (RHV) disease is an autoimmune disease affecting 0.49% of the population in developing countries (6). It is a major problem in sub-Saharan Africa, South Asia and Oceania with an estimated global incidence of 33.4 million cases and 319,400 deaths (7). Heart valve inflammation is thought to be triggered by group A streptococcal pharyngitis and in 3.6% this is followed by acute rheumatic fever (8). Rheumatic fever, if left untreated, can develop into rheumatic heart disease characterised by chronic inflammation, neovascularisation and mild calcification (9). The mitral valve is universally affected, however, concomittant aortic valve disease increases with age (8) and aortic valve pathology is understudied despite its clinical significance. Calcification in rheumatic patients is thought to be actively regulated, not simply dystrophic and warrants investigation.

Despite having different underlying aetiologies, inflammation, remodelling, valvular damage and calcification are common end points in BAV and RHV. The phenotypic changes in the valve interstitial cells (VICs) and valve endothelial cells (VECs) populations in BAV and RHV are poorly defined. Transforming growth factors (TGFs), which exist in three isoforms, TGFβ1, TGFβ2, and TGFβ3, have been implicated to play a role in a number of cardiovascular diseases, including the development of calcific aortic valve disease (10, 11). In BAV, circulating TGFβ1/endoglin has been shown to be upregulated (12, 13), while in RHV, TGFβ1 levels are raised in endothelial cells and SM cells of the vessels, in the perivascular interstitial cells and stroma of the valves as well as α-SMA positive cells in fibrotic areas cusp tissue and in the left atrial appendage of patients in chronic atrial fibrillation (14, 15). One important role for TGFs, together with increased mechanical strain, inflammatory cytokines and activation of Notch1, is to drive the process of endothelial to mesenchymal transformation (EMT) (16–20), which mediates the de-differentiation of endothelial cells into mesenchymal cells. This process has been implicated in the increased number of α-SM actin positive cells in an ovine model of functional mitral valve disease (21).

We and others have previously shown that differentiated cells are present in human calcified valves in the form of myofibroblasts and smooth muscle (SM) cells (22). We hypothesise that the native cell population in BAV and RHV undergo a similar transition process with the expression of SM markers as in aortic valve calcification which can be driven by the effects of TGFs.

## Materials and Methods

In total, 8 BAV cusps (mean age 26 years, age range 4–31, two females and six males), 7 RHV cusps (mean age 31 years, age range 7–38, one female and six male) and 12 normal aortic valve cusps (mean age 48 years, age range 36–55, eight male, two female) were used for immunocytochemistry and for cell isolation and culture. In total, 7 of the BAVs were calcified, one was fused but not calcified and 4 of the RHVs were calcified.

### Cell Isolation and Culture

Healthy human aortic valve cusps were excised and washed in PBS. The valve cusps were incubated in a collagenase solution (Type A, 0.15% w/v; Roche, Life Sciences, South San Francisco, CA, United States) for 10 mins at 37°C under a forceful agitation to remove the VECs. After centrifugation of the solution containing the VECs, the resulting VEC pellets were resuspended in media and plated out in gelatin-coated tissue culture flasks. VECs were grown until confluent in endothelial media, defined as Endothelial Cell Growth Medium 2 (ECGM; PromoCell, Heidelberg, Germany) containing 150 U/ml penicillin/streptomycin (P/S; Sigma Aldrich, Dorset, UK), 2 mM endothelial cell growth supplement, and 20% heat-inactivated fetal calf serum (FCS; Helena Biosciences, Sunderland, United Kingdom). VECs were phenotyped using flow cytometry with antibodies against CD31 and α-SMA (Dako) and cultures with >95% VEC purity were used.

All human studies have been approved by the North London Research Ethics Committee (Ref 10H0724818). These studies have been performed in accordance with the ethical standards laid down in the 1964 Declaration of Helsinki and its later amendments. All donors gave their written informed consent prior to their inclusion in the study.

### Immunochemistry

Valves were washed with PBS, fixed in 10% formal saline for 24 h, washed in distilled water and immersed in EDTA for 2 weeks at 37°C after which processing for paraffin sections was carried out. Five micrometer thick paraffin wax sections of decalcified human bicuspid and rheumatic valve tissue were dewaxed and rehydrated into water, washed in phosphate buffered saline (PBS) for 5 mins. Consecutive (adjacent) sections were used to allow regional staining patterns to be correlated with all the markers. The slides were immersed in 0.1 M citrate buffer (pH 6) and microwaved for 10 mins before blocking for endogenous peroxidases using 0.3% hydrogen peroxide in PBS. Sections were washed twice in PBS and blocked using 3% bovine serum albumin (w/v) (BSA) in PBS containing 1% v/v Tween-20 followed by staining for: α-smooth muscle actin (α-SMA), smooth muscle myosin heavy chain (SM-MHC), calponin, SM22, CD31, vimentin (all Dako), Runx2 (Abcam), myocardin (Covalab), MRTF-A (Santa-Cruz), fibronectin (Actis), PCNA (Biotechnology), HIF1α (Novus), GAPDH (Chemicon), TGFs (R&D), and TGF receptors (Thermofisher, Hemel Hempstead, UK). Negative controls consisted of 3% BSA in PBS containing 1% v/v Tween 20, isotype controls for the monoclonals and rabbit serum for the polyclonals. Primary antibodies were then removed by washing the sections three times in PBS followed by a second layer of biotinylated goat anti-mouse or swine anti-rabbit immunoglobulins (IgG-Vector laboratories) in PBS. Sections were then washed three times in PBS before 1 h incubation with Avidin-Biotin Complex ABC-Vector laboratories). Reactivity was detected using diaminobenzidine tetrahydrochloride (DAB tablets-Sigma) (25 mg/ml) and hydrogen peroxide (0.01% w/v). Sections were then counter stained with Mayers haematoxylin and viewed on Ziess Axioskop microscope. Photomicrographs were taken using Nikon DMX1200 camera.

### Immunofluorescence

Valve endothelial cells were seeded onto pre-coated gelatin or fibronectin coverslips and cultured for 10 days with or without TGFs. For immunofluorescent staining, coverslips were washed two times with PBS, fixed in 4% formaldehyde solution (Sigma Aldrich, Dorset, UK) for 10 mins and washed three times with PBS to remove the fixative solution. The coverslips were permeabilised with Triton-X-100 (0.5% v/v) for 3 mins and blocked for 30 min with BSA (3% w/v) and then incubated with primary antibodies at RT for 1 h. After thorough washing in PBS-Tween (PBS-T; Sigma Aldrich, Dorset, UK, 0.1% v/v), coverslips were stained with FITC-conjugated secondary antibodies (Invitrogen, Inchinnan, UK) for 1 h at room temperature and subsequently with DAPI for 10 mins to visualise the cell nuclei. Coverslips were washed with PBS-T, mounted on glass slides in Permafluor aqueous mounting fluid (Beckman Coulter, Fullerton, CA, United States) and analysed with confocal imaging technology.

### Transforming Growth Factors-β Treatment of Valve Endothelial Cells

Valve endothelial cells were grown on coverslips coated with 1% gelatin or 10 μg/mL fibronectin, serum-starved in DMEM containing 0.4% FCS for 24 h before being treated with 10 ng/ml of TGFβ1, TGFβ2, and TGFβ3 (R&D) for 10 days. Cells were washed with PBS and fixed in 4% paraformaldehyde for 10 mins. Staining was carried out as above.

### Ethics

This study was approved by the Royal Brompton hospital ethics review board and informed consent was obtained from the subjects.

### Statistics

**FIGURE 1:**
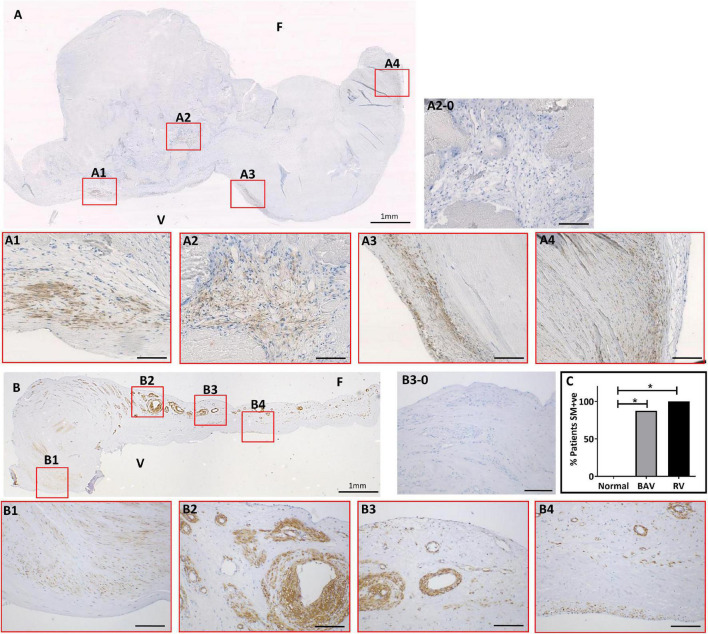
The expression of smooth muscle (SM) markers in a bicuspid aortic valve (BAV) **(A)** and a rheumatic heart valve (RHV) cusps **(B)**. Expanded panels in red boxes show staining patterns from consecutive sections with negative control **(A2-0)**, SM22 **(A1)**, α-SMA **(A2)**, calponin **(A3)**, and SM-myosin **(A4)**. Expanded panels in red boxes show staining patterns from consecutive sections of B with negative control **(B3-0)**, SM22 **(B1)**, α-SMA **(B2)**, calponin **(B3)**, and SM-myosin **(A4)**. Panel **(C)** shows graph showing the incidence of aberrant SM +ve staining in BAV and RHV cusps. F and V represent fibrosal and ventricularis side, respectively. Scale bar in expanded panels represents 200 μm. *p < 0.005.

**FIGURE 2:**
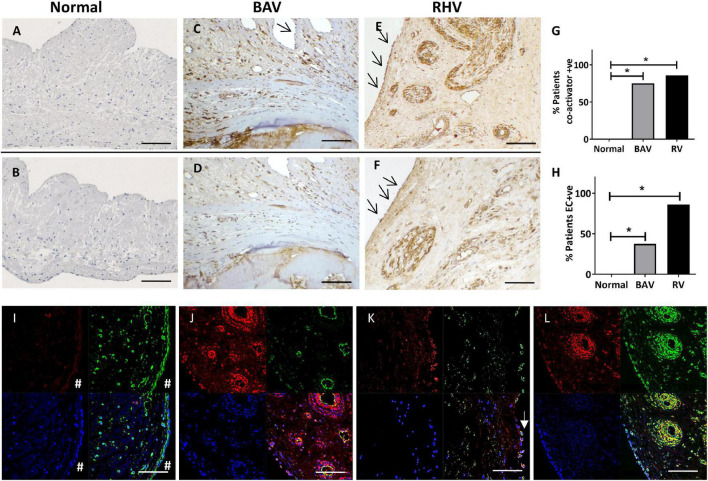
The expression of co-activators myocardin **(A,C,E)** and MRTF-A **(B,D,F)** in normal, BAV and RHV cusps. Graph showing the incidence of BAV and RHD cusps positive for aberrant co-activator expression **(G)** and aberrant expression of co-activators in valve endothelial cells (VECs) **(H)**. Panels **(I–L)** show colocalization of markers in bottom right panels. No expression of myocardin (red) in a normal cusp in VICs or VECs (_#_), vimentin (green) present in both cell types **(I)**. RHV valve showing myocardin (red) in the endothelial cells and SM cells of their vasculature, (CD31, green) of the vessels and colocalisation in endothelial cells **(J)**. Some surface VECs of a BAV showing co-expression of myocardin (red) with CD31 (green) **(K)**. RHV showing myocardin (red) and SM-MHC (green) in SM of vasculature and some VICs (left side of vessels) **(L)**. Blue is DAPI staining. Scale bar represents 200 μm. *p < 0.001.

All data was tested for normality and appropriate tests were applied. A fisher’s exact test was performed on actual numbers for Figures 1C, 2G,H, one way ANOVA and Kruskal-Wallis were used to test for significance using GraphPad Prism 5 and a *p* value of <0.05 was considered significant.

## Results

### Bicuspid Aortic Valves and Rheumatic Heart Valves Show Aberrant Expression of Smooth Muscle Markers

The expression of SM cells is localised to the base of the ventricularis in normal human cusps (Supplementary Figure 1). Occasionally a few SM cells, by their expression of SM-MHC, can be seen in the region from the base to the central region of the cusps in the ventricularis but hardly any SM cells are detected in the region from the central part of the cusps to the co-apting edge. These SM cells in normal valves express early SM markers such as α-SMA and SM22 and also late SM marker SM-MHC.

The expression of α-SMA, calponin, SM22 and SM-MHC showed mirrored patterns of staining in the same cells and regions of each valve using consecutive sections. This staining was present in clusters of cells of varying numbers predominantly around calcified nodules in BAVs but also distal to calcified zones (Figure 1A). The co-apting edges of the BAVs were markedly thickened and calcified and showed this aberrant expression more frequently than other regions of the cusp. The fibrosa and spongiosa also showed small clusters of cells expressing SM-MHC and other SM markers distal to the calcified region without any signs of calcification or thickening. A band of cells staining positive for SM markers was observed in the fibrosa in 5 BAVs. Cells positive for early and late SM markers were increased in number and present in an atypical, spatiotemporal way in 7 out of 8 of the BAVs (those that were calcified, not in the fused BAV) compared to none of the normal controls (*p* < 0.005; Figure 1C).

Early and late SM markers showed an extensive increase and atypical expression in all of the RHVs compared to none of the normal controls (*p* < 0.005; Figure 1C). This was present in a patchy manner without any specific pattern and present in all the layers and from the base to co-apting edge (Figure 1B). A high percentage VICs in the cusps stained positive for SM-MHC and other SM markers. Five of the 7 RHVs revealed the presence of many of neo-vessels which showed expression of early and late SM markers in their vasculature and endothelial lining. Significantly higher numbers of RHVs showed increased SM staining compared to normal controls (*p* < 0.005). There was no significant difference between the numbers of BAVs and RHVs showing increased SM staining (Figure 1C).

### Bicuspid Aortic Valves and Rheumatic Heart Valves Show Expression of Smooth Muscle Co-activators

The SM co-activators, myocardin (Figure 2A) and MRTF-A (Figure 2B) showed no expression in normal cusps except to the base of the ventricularis mirroring the localisation of the SM cells (Supplementary Figure 1).

In total, 6 out of 8 BAVs showed an atypical expression of myocardin (Figures 2C,G) and MRTF-A (Figures 2D,G) which was co-localised to the pattern of the SM markers in their previous sequentially cut sections compared to none of the controls (*p* < 0.001). This staining was predominantly around the calcified zones and the co-apting edges but also present distally. Their expression was also present in all three layers of the valve.

In total, 6 out of 7 RHVs showed an increased atypical pattern of expression of myocardin (Figures 2E,G) and MRTF-A (Figures 2F,G) compared to the normal controls (*p* < 0.001). This pattern mirrored that of the SM markers in their previous sequentially cut sections with patchy positive staining of VICs in all the layers, from the base to the co-apting edge. The vasculature of all the neo-vessels was positively stained for both markers. There was no significant difference between the numbers of BAVs and RHs showing co-factor staining (Figure 2G).

### Valve Endothelial Cells From Bicuspid Aortic Valves and Rheumatic Heart Valves Aberrantly Express Smooth Muscle Markers and Co-activators

In total, 3 out of 8 BAVs showed VECs that were positive for SM markers and co-activators (myocardin and MRTF-A) compared to none of the normal controls (*p* < 0.01). These positive VECs were partly on the valve surface, but mostly on ECs lining small neovessels (Figures 2C,D,H).

All of the RHVs showed some regions of positive VEC staining for SM markers and co-activators compared to none of the normal controls (*p* < 0.0001). Regions of the endothelium on both aortic and ventricular surfaces of the cusps, and ECs lining the small to large neo-vessels were found to be positive for SM markers and co-activators in RHVs (Figures 2E,F,H).

There was no significant difference between the numbers of BAVs and RHVs staining positive for SM markers and co-activators. The pattern of staining for myocardin and MRTF-A in VECs and VICs mostly overlapped in each valve for BAVs and RHVs as could be seen with staining using consecutive sections (Figures 2C,D).

To definitively identify the phenotype of the cells expressing the co-activators, co-localisation staining was performed. Normal valves showed no staining for myocardin in the VICs or VECs (Figure 2I). RHVs showed myocardin in the endothelial cells of the vessels (Figure 2J) as well as in the SM cells of their vasculature (Figures 2J,L) and both BAVs and RHVs showed expression in VICs expressing SM-MHC (Figure 2L). Some surface VECs (both ventricular and fibrosal sides) of both BAVs and RHVs showed co-expression of myocardin (Figure 2K).

### Valve Endothelial Cells Co-express Smooth Muscle Markers and Markers of Calcification

**FIGURE 3:**
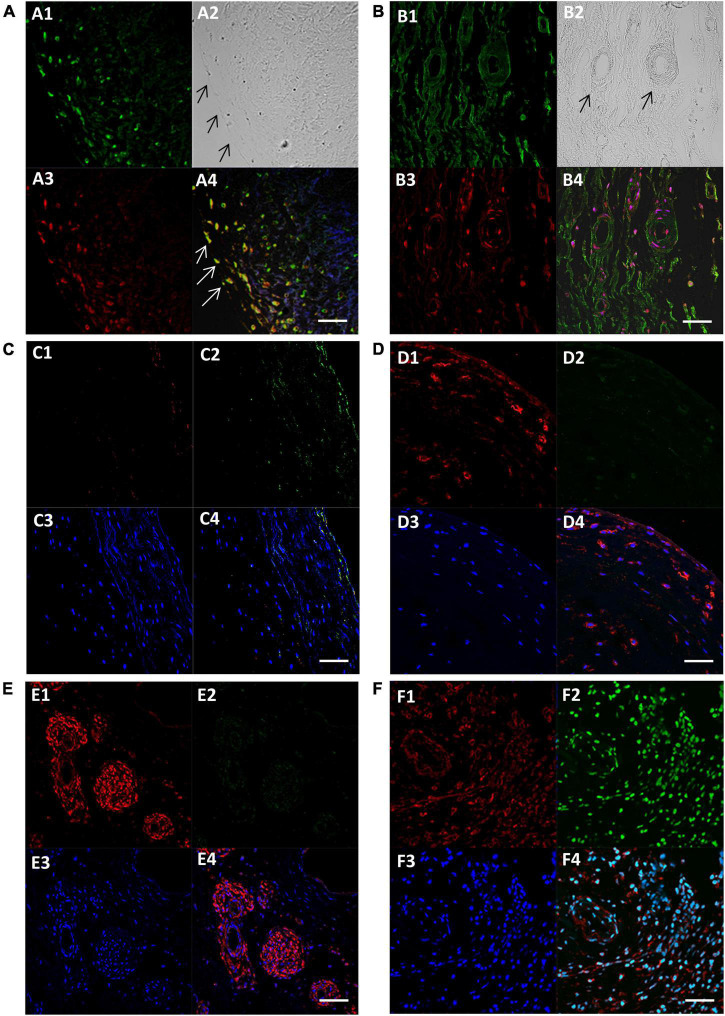
Fluorescent images showing expression of SM-myosin **(A1)**, phase contrast image of the same location showing the edge of the cusp on the left **(A2)**, expression of Runx2 **(A3)**, and co-localisation of SM-myosin and Runx2 **(A4)** in a BAV cusp. Expression of SM-myosin **(B1)**, phase contrast **(B2)**, Runx2 **(B3)**, and co-localisation of SM-myosin and Runx2 **(B4)** in a RHV cusp. Expression of myocardin **(C1–F1)**, PCNA **(C2–F2)**, DAPI **(C3–F3)** and co-localisation of all three respective previous panels **(F1–F4)** in a normal control (**C** panel), BAV cusp (**D** panel), RHV cusp (**E** panel), and tumour tissue (**F** panel) as positive control for PCNA. Scale bar represents 50 μm.

We questioned whether the VECs that expressed SM markers also co-expressed markers of calcification. We observed that some VECs at the surface and just under the surface of 5 BAVs were able to co-express SM markers and Runx2 (Figures 3A1–4). Four RHVs showed more co-expression of SM and Runx2 in VECs of the neovessels than the cusp surfaces (Figures 3B1–4).

### Cells Expressing Smooth Muscle Markers and Co-activators Are Not Proliferative

The presence of cells expressing SM markers within the BAVs and RHVs provoked the question of whether these cells are contractile or synthetic and we assessed this by co-staining for proliferative markers PCNA and Ki67 with myocardin. We did not observe any PCNA or Ki67 (not shown) staining that colocalised with the SM markers (Figures 3C–F).

### Assessment of Hypoxia Inducible Factor 1α and Correlation With Smooth Muscle Markers

**FIGURE 4:**
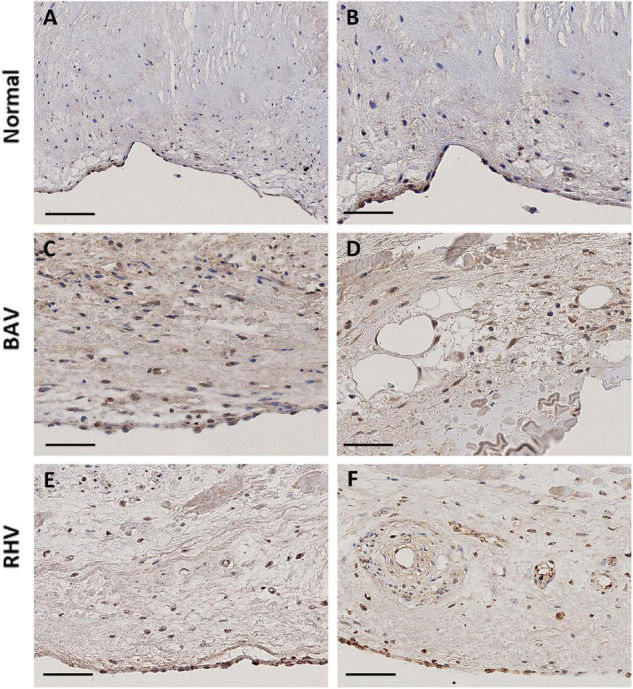
The expression of HIF1α in a normal cusp **(A,B)**, BAV **(C,D)**, and RHV **(E,F)**. Scale bar is 100 μm in panel **(A)** and 50 μm in all other panels.

Both BAV and RHV were significantly thickened with BAV having a maximal average thickness of 2.97 ± 1.2 mm and RHV, 2.05 ± 0.56 mm. Cells in such thick BAVs and RHVs may become hypoxic and initiate pathways to induce hypoxia inducible factor 1α (HIF1α). The expression of HIF1α was correlated with SM markers by analysing HIF1α in all groups. Normal cusps showed rare nuclear HIF1α-positive VICs, however, of the seven normal cusps analysed, three showed some ventricular VEC nuclear HIF1α positivity with the occasional nuclear positive VIC (Figures 4A,B). There was no HIF1α expression in the BAV that was not calcified despite being thickened. Of the remaining seven calcified, thickened BAVs, four showed many VICs with nuclear HIF1α expression (Figure 4C) around and distal to calcified regions, with numbers of positive cells far outnumbering those expressing SM markers and the remaining BAVs showed occasional nuclear positive VICs. There was occasional surface (both sides) nuclear VEC HIF1α expression in 2 BAVs (Figure 4C) and the endothelial cells of vessels were marginally positive (Figure 4D). Three of the RHVs showed no expression of HIF1α. The other four showed strong nuclear expression of HIF1α on some fibrosal and ventricular VEC (Figures 4E,F) and weaker expression in the vasculature of the vessels (Figure 4F). Only 1 RHV showed endothelium positivity of the vessels and the same RHV showed some HIF1α in VICs. The pattern of HIF1α did not correspond to the SM marker expression in BAVs and RHVs.

### Smooth Muscle Markers and Co-activators Are Modulated by Transforming Growth Factors and Substrate Coating in Valve Endothelial Cells

**FIGURE 5:**
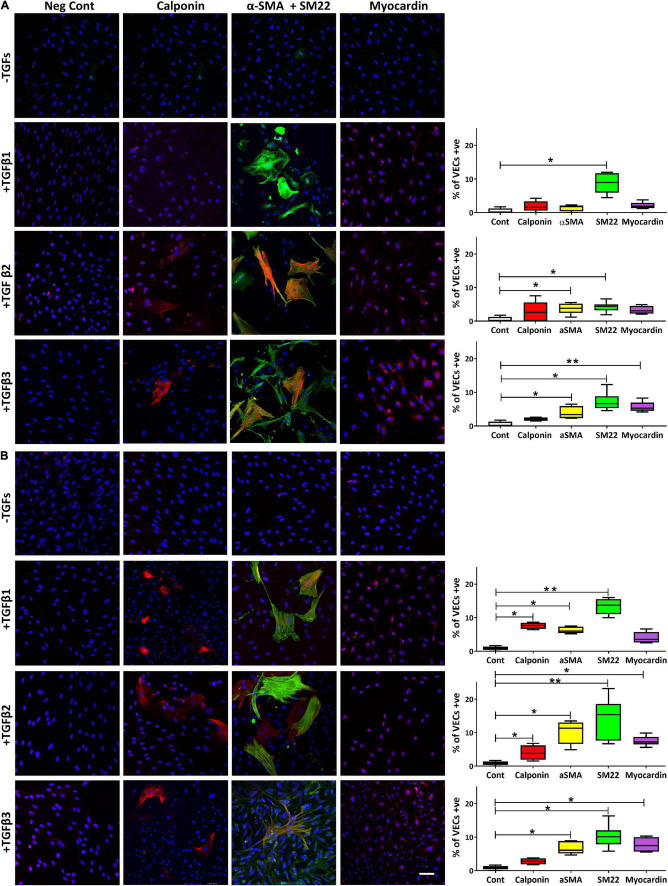
The expression of calponin, α-SMA and SM22 merged, and myocardin in VECs plated on gelatin **(A)** and on fibronectin **(B)** and treated with TGFs. Scale bar represents 50 μm. *p,0.05, **p < 0.01.

We have previously shown that TGFβ1 was able to upregulate the expression of SM markers and MRTF-A in VICs so we sought to address whether TGFβs could modulate their expression in VECs as well as assessing the role of surface coating with gelatin and fibronectin. On gelatin coated slides, VECs treated with TGFβ1 only showed a significant expression of SM22 (*p* < 0.05), treated with TGFβ2 showed a significant expression of αSMA (*p* < 0.05) and SM22 (*p* < 0.05) and treated with TGFβ3 showed a significant expression of αSMA (*p* < 0.05), SM22 (*p* < 0.05) and myocardin (*p* < 0.01; Figure 5A).

However, when VECs were cultured on fibronectin, TGFβ1 was able to significantly increase calponin (*p* < 0.05), α-SMA (*p* < 0.05) and SM22 (*p* < 0.01); TGFβ2 was able to significantly increase calponin (*p* < 0.05), αSMA (*p* < 0.05), SM22 (*p* < 0.01) and myocardin (*p* < 0.05); TGFβ3 was able to significantly increase αSMA (*p* < 0.05) and SM22 (*p* < 0.05) and myocardin (*p* < 0.05; Figure 5B).

### Fibronectin Is Upregulated in Bicuspid Aortic Valves and Rheumatic Heart Valves

**FIGURE 6:**
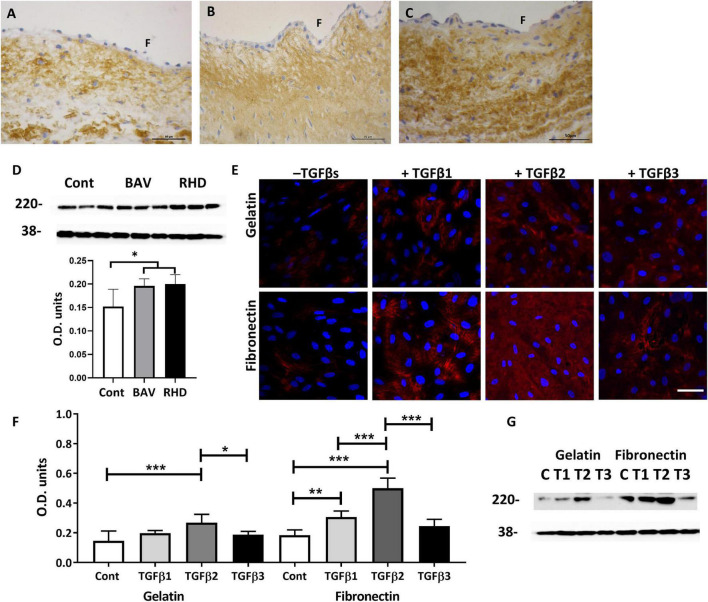
The expression of fibronectin on a normal **(A)** BAV, **(B)** RHV, **(C)** cusp, and quantitative levels **(D)**. The expression of fibronectin by VECs plated on gelatin (top panel) and on fibronectin (bottom panel) and treated with TGFs **(E)**. Graph showing the level of expression of fibronectin by VECs when plated on gelatin or fibronectin and treated with TGFs **(F)** and the corresponding Western blot showing levels of fibronectin at 220 kD and the housekeeping protein GAPDH at 38 kD **(G)**. Panel **(F)** represents the fibrosal side. Scale bar in panel **(A–C)** is 50 μm, in panel **(D)** is 100 μm. *p,0.05, **p < 0.01, ***p < 0.001.

As the response of VECs to upregulate SM markers and myocardin was more pronounced when treated with TGFβs cultured on fibronectin, we questioned whether the expression of fibronectin was dysregulated in BAVs and RHVs. Normal cusps showed moderate staining in the fibrosa and ventricularis with reduced and patchy staining in the spongiosa (Figure 6A). 6/8 BAVs (Figure 6B) and 5/7 RHVs (Figure 6C) showed increased intensity of staining for fibronectin in the fibrosa, ventricularis and in the basal lamina with increased patchy staining of the spongiosa. Quantitation showed significantly increased levels of fibronectin in BAVs and RHVs (Figure 6D), *p* < 0.05.

### Transforming Growth Factor βs Enhance Fibronectin Expression by Valve Endothelial Cells Preferentially Plated on Fibronectin

As fibronectin was increased in BAVs and RHVs, we questioned whether TGFs were able to differentially upregulate fibronectin produced by VECs, whether plated on gelatin or fibronectin. Untreated VECs plated on gelatin and fibronectin showed a similar low intensity of staining and level for fibronectin (Figures 6E–G). TGFβ1 was able to significantly upregulate the expression of fibronectin by VECs but only when plated on fibronectin (*p* < 0.01). TGFβ2 was able to significantly upregulate the expression of fibronectin by VECs plated on gelatin and fibronectin (*p* < 0.001). Increased expression of fibronectin was observed within the VECs and in the extracellular spaces (Figure 6E). However, TGFβ3 was unable to upregulate fibronectin by VECs either on gelatin or fibronectin coated plates (Figures 6F,G).

### Transforming Growth Factor β1, TGFRI, and TGFRII Are Upregulated in Bicuspid Aortic Valves and Rheumatic Heart Valves

**FIGURE 7:**
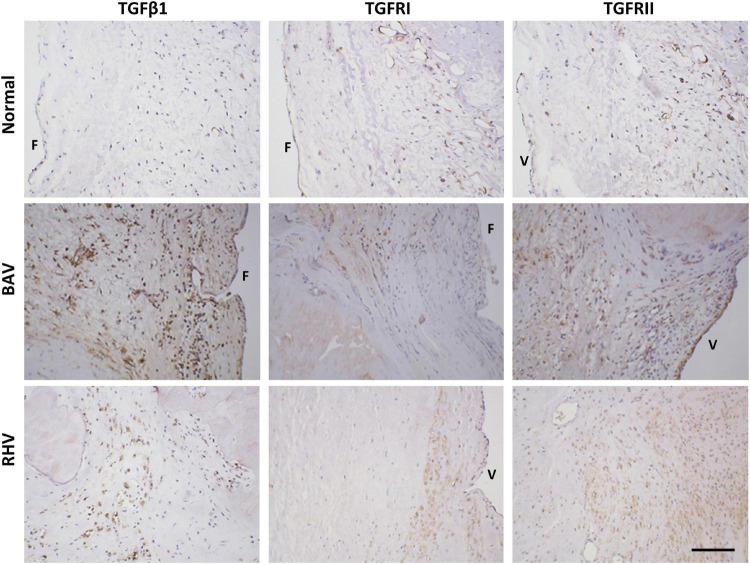
The expression of transforming growth factor β1 (TGFβ1), TGFR1, and TGFRII in normal, BAV and RHV cusps. F and V represent the fibrosal and ventricularis side, respectively. Scale bar is 200 μm.

There was no to very little expression of TGFβ1 in normal cusps, however, BAVs and RHVs showed greatly increased numbers of VICs staining positive for TGFβ1. The antibodies for TGFβ2 and TGFβ3 were found not be suitable for human valve tissue in paraffin sections. Both TGFRI and TGFRII were found to be expressed in up to 50% of normal VICs, however, some regions in cusps from BAVs and RHVs showed 100% expression of TGFRII (Figure 7).

## Discussion

Calcification is expedited in BAVs and occasionally present in aortic RHVs and this is partly driven by the native cell population and partly through inflammatory cells, mediators and disturbed flow patterns. We have shown that both the VECs and VICs of BAVs and RHVs exhibit an atypical expression of SM markers and the co-activators myocardin and MRTF-A. The expression of these markers can be induced in VECs by TGFβs, an effect that is enhanced when the cells were cultured on fibronectin. Fibronectin expression in the diseased groups was found to be enhanced within the cusp layers as well as in the basement membrane providing a conducive environment for VEC differentiation. The elevated expression of TGFβ receptors concomittant with enhanced fibronectin expression in BAVs and RHVs would facilitate the signalling by TGFs in BAVs and RHVs.

We have previously shown an upregulated and dysregulated spatial expression of SM markers and co-activators, myocardin and MRTFs, in both the VICs and VECs of calcified tricuspid valves (22). α-SMA has been shown previously in calcified valves and the valvular cells were ascribed a myofibroblastic phenotype, however, when these cells also co-express SM-specific markers such as SM-myosin heavy chain, SM master co-activator myocardin and structural charactersitics of SM cells, these cells are best described as SM cells. A minority of cells expressing α-SMA did not express the SM-MHC in our previous study and in this study and are thus myofibroblasts. It is quite plausible that the VICs expressing the early SM marker SM22 without the late marker SM-MHC are designated myofibroblasts before they go on to expressing the late SM markers and co-activators. Remarkably there is scarce published data on the phenotypic changes occuring in human BAVs. One study reported no difference in the expression of α-SMA between BAV and TAV VICs in culture (23), however, it is known that VICs differentiate *in vitro* (24, 25) and this difference would have been nullified by *in vitro* culture under their conditions. Similarly, the expression of SM markers and co-activators have not been previously shown in VICs and VECs in RHV.

The SM marker and co-activator expressing cells were shown not to be proliferative by the absence of PCNA in myocardin positive cells and are most likely to be of the contractile type as they strongly expressed SM-MHC and myocardin. Overexpression of myocardin has been shown to inhibit cell cycle progression (26) and both MRTF-A and -B have anti-proliferative effects (27). The differentiation of valvular cells into a contractile nature, as we have shown previously in tricuspid calcified valves (22) may be a compensatory mechanism to overcome the stiffening entailed during the disease process.

Valve endothelial cell differentiation into SM types has been previously reported by us (22) and endothelial cells have been shown to have transdifferentiation potential into the SM phenotype by inducing the expression of myocardin (28) and cyclic strain (29).

Normal aortic cusps have a mean thickness of 0.60 ± 0.21 mm and this allows diffusion of oxygen without the need for a vascular system (30). Only 4 BAVs and 1 RHV showed an increase in HIF1α in VICs despite being significantly thicker. The abundance of HIF1α in BAVs did not result in significant neoangiogenesis in the central layers though vessels were observed in the outer layers of 3 BAVs. Surprisingly, 4 of the 7 RHVs showed strong HIF1α expression in the surface VECs where hypoxic conditions would not be expected. There is significance in the expression of HIF1α in the SM cells of the vasculature as HIF1α has been shown to play a role in phosphate-induced vascular SM cell calcification (31). We have previously documented a similar pattern of expression of HIF1α in rheumatic mitral VICs, surface VECs and in vessels and the induction of HIF1α by hypoxic conditions (32). HIF1α can also be induced by disturbed flow (33) and TNFα (34) and has been shown in stenotic aortic valves (35). The role of HIF1α warrants further investigation in diseased cusps.

Proinflammatory cytokines play a role in the progression and maintenance of valvular cell differentiation and TGFβ1 has been shown to be present in degenerative calcific aortic stenotic cusps. Contradictingly, TGFβ1 was shown not to be increased in the media from BAV or rheumatic valves (36) but in another study found to be highly present in 70% of rheumatic mitral valves (14). Elevated TGFβ1 can also result from polymorphisms in the TGF1 gene and it was shown that RV patients have a lower frequency of TGFβ1 C?T (509) genotype and a higher frequency of T?C (869) allele with the latter polymorphism resulting in raised TGFβ1 (37).

Transforming growth factor β1-mediated endothelial to mesenchymal transformation (EMT) has been shown to correlate with enhanced expression of laminin and fibronectin (38). TGF-β1 is extremely important in inducing the differentiation of VICs to myofibroblasts (25, 39). The expression of all three isoforms of TGFβs will play a significant role in the differentiation of adult VECs undergoing EMT and further differentiating to express SM markers. All three isoforms have been shown to induce EMT in human microvascular ECs with the effect of TGFβ2 being most pronounced (40). The presence of SM markers and co-activators in VECs of BAVs and RHVs is indicative of an on-going EMT process in these cells. Cues from specific ECM components can also play a role in the EMT process and fibronectin was shown to be present at early stages of EMT and appeared as a progressively expanding gradient of material with the greatest density nearer the myocardium in the AV canal and outflow tract (41). Fibronectin is abundant in the ECM through which endocardial-mesenchymal cells migrate as they begin formation of the cushion tissue (41) and it is a key component of the basement membrane on which the VECs reside. EMT is a crucial process for valve development but is not restricted to embryonic development as adult ovine mitral VECs have been shown to undergo EMT (42).

We have previously shown the spatial expression of fibronectin in normal aortic valves (43) and now show that BAVs and RHVs demonstrated an enhanced expression of fibronectin within the valves and at regions of the basement membrane. This increased expression of fibronectin is expressed by VICs that have become activated in response to injury (44) and by the VECs. The combination of increased fibronectin and TGFs can synergistically provide the signals for VEC EMT and drive the differentiation process further to express SM markers and co-activators. Alterations in ECM composition are known to affect EMT with collagen IV and fibronectin promoting EMT in ovine mitral VECs (45) and high levels of chondroitin sulphate have been shown to induce the highest rates of EMT in porcine VECs (46).

Inhibiting the pathological differentiation of resident cells of the valve is key in the therapeutic intervention and prevention of calcification. The high propensity of SM cells to calcify and result in atherosclerotic lesions suggests that this SM-type differentiation would be a key step for intervention. However, it remains to be determined what proportion of resident cells differentiate to osteoblastic-type cells through the SM phenotype and whether some cells can bypass this transition.

This report documents the atypical expression of SM markers and co-activators in BAVs and RHVs and shows that the TGFs can preferentially increase these markers dependent on substrate transformation in terms of composition and stiffness. It is likely that the SM-positive cells in the diseased BAV and RHV are derived from endothelial cells, via an EMT process, from differentiation of the VIC population and possibly from infiltrating cells. The significance of the SM cells may be that they have a greater propensity to calcify. The elevated expression of fibronectin and TGF receptors augments this efficacy of the signalling pathways that stimulate the VECs to express SM markers. Further studies are warranted to ascertain the pathological contribution of SM cells in the valves and whether strategies aimed at blocking this transformation would prove beneficial as a therapeutic intervention. This study has provided an insight into the phenotypic changes in the valve cell population that occur in BAVs and RHVs. Understanding the contribution that specific cell phenotypes make to valve disease represents an important step toward the development of novel strategies to control or prevent the progression of the disease.

## Data Availability Statement

The original contributions presented in the study are included in the article/Supplementary Material, further inquiries can be directed to the corresponding author.

## Ethics Statement

The studies involving human participants were reviewed and approved by Royal Brompton and Harefield Ethics Committee. The patients/participants provided their written informed consent to participate in this study.

## Author Contributions

NL designed and executed the work, analysed the data, wrote the manuscript, and proofread. PS and AM performed some of the experiments. MY and AC designed, wrote, and proofread. All authors contributed to the article and approved the submitted version.

## Conflict of Interest

The authors declare that the research was conducted in the absence of any commercial or financial relationships that could be construed as a potential conflict of interest.

## Publisher’s Note

All claims expressed in this article are solely those of the authors and do not necessarily represent those of their affiliated organizations, or those of the publisher, the editors and the reviewers. Any product that may be evaluated in this article, or claim that may be made by its manufacturer, is not guaranteed or endorsed by the publisher.

## Abbreviations

BAV: bicuspid aortic valve
RHV: rheumatic heart valve
SM: smooth muscle
TGF: transforming growth factor
MRTF-A: myocardin related transcription factor A
VIC: valve interstitial cell
VEC: valve endothelial cell
PCNA: proliferating nuclear cell antigen
HIF1α: hypoxia inducible factor 1α.

## References

[1] Tutar E Ekici F Atalay S Nacar N. The prevalence of bicuspid aortic valve in newborns by echocardiographic screening. *Am Heart J.* (2005) 150:513–5. 10.1016/j.ahj.2004.10.036 16169333

[2] Siu SC Silversides CK. Bicuspid aortic valve disease. *J Am Coll Cardiol.* (2010) 55:2789–800. 20579534 10.1016/j.jacc.2009.12.068

[3] Masri A Svensson LG Griffin BP Desai MY. Contemporary natural history of bicuspid aortic valve disease: a systematic review. *Heart.* (2017) 103:1323–30. 10.1136/heartjnl-2016-309916 28490615

[4] Garg V Muth AN Ransom JF Schluterman MK Barnes R King IN Mutations in NOTCH1 cause aortic valve disease. *Nature.* (2005) 437:270–4. 10.1038/nature03940 16025100

[5] Laforest B Andelfinger G Nemer M. Loss of Gata5 in mice leads to bicuspid aortic valve. *J Clin Invest.* (2011) 121:2876–87. 10.1172/JCI44555 21633169 PMC3223824

[6] Zuhlke LJ Steer AC. Estimates of the global burden of rheumatic heart disease. *Glob Heart.* (2013) 8:189–95. 25690495 10.1016/j.gheart.2013.08.008

[7] Watkins DA Johnson CO Colquhoun SM Karthikeyan G Beaton A Bukhman G Global, regional, and national burden of rheumatic heart disease, 1990-2015. *N Engl J Med.* (2017) 377:713–22. 10.1056/NEJMoa1603693 28834488

[8] Carapetis JR Beaton A Cunningham MW Guilherme L Karthikeyan G Mayosi BM Acute rheumatic fever and rheumatic heart disease. *Nat Rev Dis Primers.* (2016) 2:15084. 27188830 10.1038/nrdp.2015.84 PMC5810582

[9] Xiao F Zheng R Yang D Cao K Zhang S Wu B Sex-dependent aortic valve pathology in patients with rheumatic heart disease. *PLoS One.* (2017) 12:e0180230. 10.1371/journal.pone.0180230 28662157 PMC5491156

[10] Agrotis A Kalinina N Bobik A. Transforming growth factor-beta, cell signaling and cardiovascular disorders. *Curr Vasc Pharmacol.* (2005) 3:55–61. 10.2174/1570161052773951 15638782

[11] Jian B Narula N Li QY Mohler ER III Levy RJ. Progression of aortic valve stenosis: TGF-beta1 is present in calcified aortic valve cusps and promotes aortic valve interstitial cell calcification via apoptosis. *Ann Thorac Surg.* (2003) 75:457–65; discussion 65–6. 10.1016/s0003-4975(02)04312-6 12607654

[12] Hillebrand M Millot N Sheikhzadeh S Rybczynski M Gerth S Kolbel T Total serum transforming growth factor-beta1 is elevated in the entire spectrum of genetic aortic syndromes. *Clin Cardiol.* (2014) 37:672–9. 10.1002/clc.22320 25113270 PMC6649456

[13] Rocchiccioli S Cecchettini A Panesi P Farneti PA Mariani M Ucciferri N Hypothesis-free secretome analysis of thoracic aortic aneurysm reinforces the central role of TGF-beta cascade in patients with bicuspid aortic valve. *J Cardiol.* (2017) 69:570–6. 10.1016/j.jjcc.2016.05.007 27298013

[14] Kim L Kim DK Yang WI Shin DH Jung IM Park HK Overexpression of transforming growth factor-beta 1 in the valvular fibrosis of chronic rheumatic heart disease. *J Korean Med Sci.* (2008) 23:41–8. 10.3346/jkms.2008.23.1.41 18303197 PMC2526480

[15] Zhang D Liu X Chen X Gu J Li F Zhang W Role of the MAPKs/TGF-beta1/TRAF6 signaling pathway in atrial fibrosis of patients with chronic atrial fibrillation and rheumatic mitral valve disease. *Cardiology.* (2014) 129:216–23. 10.1159/000366096 25376239

[16] Paranya G Vineberg S Dvorin E Kaushal S Roth SJ Rabkin E Aortic valve endothelial cells undergo transforming growth factor-beta-mediated and non-transforming growth factor-beta-mediated transdifferentiation in vitro. *Am J Pathol.* (2001) 159:1335–43. 10.1016/s0002-9440(10)62520-5 11583961 PMC1850524

[17] Balachandran K Alford PW Wylie-Sears J Goss JA Grosberg A Bischoff J Cyclic strain induces dual-mode endothelial-mesenchymal transformation of the cardiac valve. *Proc Natl Acad Sci USA.* (2011) 108:19943–8. 10.1073/pnas.1106954108 22123981 PMC3250145

[18] Farrar EJ Butcher JT. Heterogeneous susceptibility of valve endothelial cells to mesenchymal transformation in response to TNFalpha. *Ann Biomed Eng.* (2014) 42:149–61. 10.1007/s10439-013-0894-3 23982279 PMC3905205

[19] Mahler GJ Farrar EJ Butcher JT. Inflammatory cytokines promote mesenchymal transformation in embryonic and adult valve endothelial cells. *Arterioscler Thromb Vasc Biol.* (2013) 33:121–30. 10.1161/ATVBAHA.112.300504 23104848 PMC3694265

[20] Yang JH Wylie-Sears J Bischoff J. Opposing actions of Notch1 and VEGF in post-natal cardiac valve endothelial cells. *Biochem Biophys Res Commun.* (2008) 374:512–6. 10.1016/j.bbrc.2008.07.057 18647596 PMC2574620

[21] Bischoff J Casanovas G Wylie-Sears J Kim DH Bartko PE Guerrero JL CD45 expression in mitral valve endothelial cells after myocardial infarction. *Circ Res.* (2016) 119:1215–25. 10.1161/CIRCRESAHA.116.309598 27750208 PMC5215059

[22] Latif N Sarathchandra P Chester AH Yacoub MH. Expression of smooth muscle cell markers and co-activators in calcified aortic valves. *Eur Heart J.* (2015) 36:1335–45. 10.1093/eurheartj/eht547 24419809

[23] Aggarwal A Ferrari G Joyce E Daniels MJ Sainger R Gorman JH III Architectural trends in the human normal and bicuspid aortic valve leaflet and its relevance to valve disease. *Ann Biomed Eng.* (2014) 42:986–98. 10.1007/s10439-014-0973-0 24488233 PMC4364391

[24] Latif N Quillon A Sarathchandra P McCormack A Lozanoski A Yacoub MH Modulation of human valve interstitial cell phenotype and function using a fibroblast growth factor 2 formulation. *PLoS One.* (2015) 10:e0127844. 10.1371/journal.pone.0127844 26042674 PMC4456368

[25] Porras AM van Engeland NC Marchbanks E McCormack A Bouten CV Yacoub MH Robust generation of quiescent porcine valvular interstitial cell cultures. *J Am Heart Assoc.* (2017) 6:e005041. 10.1161/JAHA.116.005041 28292746 PMC5524027

[26] Milyavsky M Shats I Cholostoy A Brosh R Buganim Y Weisz L Inactivation of myocardin and p16 during malignant transformation contributes to a differentiation defect. *Cancer Cell.* (2007) 11:133–46. 10.1016/j.ccr.2006.11.022 17292825

[27] Shaposhnikov D Descot A Schilling J Posern G. Myocardin-related transcription factor A regulates expression of Bok and Noxa and is involved in apoptotic signalling. *Cell Cycle.* (2012) 11:141–50. 10.4161/cc.11.1.18499 22185759

[28] Ji H Atchison L Chen Z Chakraborty S Jung Y Truskey GA Transdifferentiation of human endothelial progenitors into smooth muscle cells. *Biomaterials.* (2016) 85:180–94. 10.1016/j.biomaterials.2016.01.066 26874281 PMC4763719

[29] Cevallos M Riha GM Wang X Yang H Yan S Li M Cyclic strain induces expression of specific smooth muscle cell markers in human endothelial cells. *Differentiation.* (2006) 74:552–61. 10.1111/j.1432-0436.2006.00089.x 17177852

[30] Yacoub MH Tsang V Sarathchandra P Jensen H Hughes S Latif N. Long-term adaptive versus maladaptive remodelling of the pulmonary autograft after the Ross operation. *Eur J Cardiothorac Surg.* (2020) 57:977–85. 10.1093/ejcts/ezaa019 32129834

[31] Mokas S Lariviere R Lamalice L Gobeil S Cornfield DN Agharazii M Hypoxia-inducible factor-1 plays a role in phosphate-induced vascular smooth muscle cell calcification. *Kidney Int.* (2016) 90:598–609. 10.1016/j.kint.2016.05.020 27470678

[32] Salhiyyah K Sarathchandra P Latif N Yacoub MH Chester AH. Hypoxia-mediated regulation of the secretory properties of mitral valve interstitial cells. *Am J Physiol Heart Circ Physiol.* (2017) 313:H14–23. 10.1152/ajpheart.00720.2016 28314761

[33] Fernandez Esmerats J Villa-Roel N Kumar S Gu L Salim MT Ohh M Disturbed flow increases UBE2C (ubiquitin E2 ligase C) via loss of miR-483-3p, inducing aortic valve calcification by the pVHL (von Hippel-Lindau protein) and HIF-1alpha (hypoxia-inducible factor-1alpha) pathway in endothelial cells. *Arterioscler Thromb Vasc Biol.* (2019) 39:467–81. 10.1161/ATVBAHA.118.312233 30602302 PMC6393167

[34] Parra-Izquierdo I Sanchez-Bayuela T Lopez J Gomez C Perez-Riesgo E San Roman JA Interferons are pro-inflammatory cytokines in sheared-stressed human aortic valve endothelial cells. *Int J Mol Sci.* (2021) 22:10605. 10.3390/ijms221910605 34638942 PMC8508640

[35] Perrotta I Moraca FM Sciangula A Aquila S Mazzulla S. HIF-1alpha and VEGF: immunohistochemical profile and possible function in human aortic valve stenosis. *Ultrastruct Pathol.* (2015) 39:198–206. 10.3109/01913123.2014.991884 25569379

[36] Kochtebane N Passefort S Choqueux C Ainoun F Achour L Michel JB Release of leukotriene B4, transforming growth factor-beta1 and microparticles in relation to aortic valve calcification. *J Heart Valve Dis.* (2013) 22:782–8. 24597398

[37] Chou HT Chen CH Tsai CH Tsai FJ. Association between transforming growth factor-beta1 gene C-509T and T869C polymorphisms and rheumatic heart disease. *Am Heart J.* (2004) 148:181–6. 10.1016/j.ahj.2004.03.032 15215809

[38] Sales VL Engelmayr GC Jr Mettler BA Johnson JA Jr Sacks MS Mayer JE. Jr Transforming growth factor-beta1 modulates extracellular matrix production, proliferation, and apoptosis of endothelial progenitor cells in tissue-engineering scaffolds. *Circulation.* (2006) 114(1 Suppl.):I193–9. 10.1161/CIRCULATIONAHA.105.001628 16820571

[39] Walker GA Masters KS Shah DN Anseth KS Leinwand LA. Valvular myofibroblast activation by transforming growth factor-beta: implications for pathological extracellular matrix remodeling in heart valve disease. *Circ Res.* (2004) 95:253–60. 10.1161/01.RES.0000136520.07995.aa 15217906

[40] Sabbineni H Verma A Somanath PR. Isoform-specific effects of transforming growth factor beta on endothelial-to-mesenchymal transition. *J Cell Physiol.* (2018) 233:8418–28. 10.1002/jcp.26801 29856065 PMC6415927

[41] Kitten GT Markwald RR Bolender DL. Distribution of basement membrane antigens in cryopreserved early embryonic hearts. *Anat Rec.* (1987) 217:379–90. 10.1002/ar.1092170409 3592264

[42] Wylie-Sears J Aikawa E Levine RA Yang JH Bischoff J. Mitral valve endothelial cells with osteogenic differentiation potential. *Arterioscler Thromb Vasc Biol.* (2011) 31:598–607. 10.1161/ATVBAHA.110.216184 21164078 PMC3210435

[43] Latif N Sarathchandra P Taylor PM Antoniw J Yacoub MH. Localization and pattern of expression of extracellular matrix components in human heart valves. *J Heart Valve Dis.* (2005) 14:218–27. 15792183

[44] Fayet C Bendeck MP Gotlieb AI. Cardiac valve interstitial cells secrete fibronectin and form fibrillar adhesions in response to injury. *Cardiovasc Pathol.* (2007) 16:203–11. 10.1016/j.carpath.2007.02.008 17637428

[45] Wang Z Calpe B Zerdani J Lee Y Oh J Bae H High-throughput investigation of endothelial-to-mesenchymal transformation (EndMT) with combinatorial cellular microarrays. *Biotechnol Bioeng.* (2016) 113:1403–12. 10.1002/bit.25905 26666585

[46] Dahal S Huang P Murray BT Mahler GJ. Endothelial to mesenchymal transformation is induced by altered extracellular matrix in aortic valve endothelial cells. *J Biomed Mater Res A.* (2017) 105:2729–41. 10.1002/jbm.a.36133 28589644

