# Supplementary material for: Atypical Expression of Smooth Muscle Markers and Co-activators and Their Regulation in Rheumatic Aortic and Calcified Bicuspid Valves
Source: Front Cardiovasc Med. 2022 Mar 17;9:793666. doi: 10.3389/fcvm.2022.793666 (PMC8968087; doi:10.3389/fcvm.2022.793666)
Supplement: Supplementary file 1 [file Image_1.pdf]

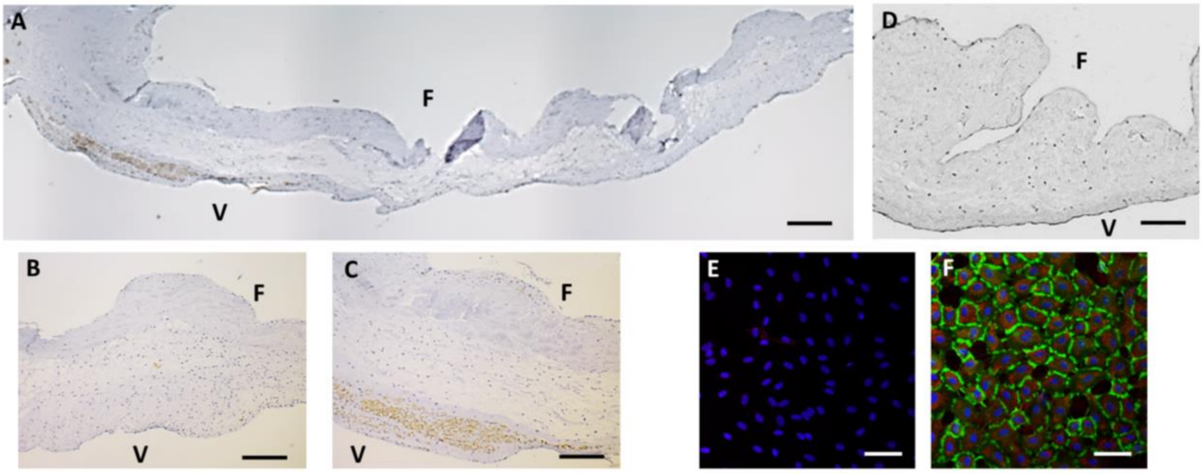

Supplementary Fig.1

The expression of SM markers in a normal aortic valve (A) stained with α-SMA (A), negative control (B) and SM-myosin (C). The expression of CD31 on a normal aortic cusp (D), negative control for cultured VECs (E) and CD31 staining for cultured VECs (F). Scale bar in A represents 200µm, in B, C and D represents 100µm and in E and F represents 50µm.
